# Predictors of Remission of Early-Onset Poststroke Depression and the Interaction Between Depression and Cognition During Follow-Up

**DOI:** 10.3389/fpsyt.2018.00738

**Published:** 2019-01-08

**Authors:** Jing Huang, Fu-Chun Zhou, Boyuan Guan, Ning Zhang, Anxin Wang, Ping Yu, Lei Zhou, Chuan-Yue Wang, Chunxue Wang

**Affiliations:** ^1^Department of Neuropsychiatry and Behavioral Neurology and Clinical Psychology, Beijing Tiantan Hospital, Capital Medical University, Beijing, China; ^2^China National Clinical Research Center of Neurological Diseases, Beijing Tiantan Hospital, Capital Medical University, Beijing, China; ^3^Center of Stroke, Beijing Institute for Brain Disorders, Beijing, China; ^4^Beijing Key Laboratory of Translational Medicine for Cerebrovascular Disease, Beijing, China; ^5^Beijing Key Laboratory of Mental Disorders, Beijing Anding Hospital, Capital Medical University, Beijing, China; ^6^Beijing Institute for Brain Disorders Center of Schizophrenia, Beijing Anding Hospital, Capital Medical University, Beijing, China; ^7^The National Clinical Research Center for Mental Disorders, Beijing Anding Hospital, Capital Medical University, Beijing, China

**Keywords:** early-onset, poststroke depression, predictors of remission, cognitive impairment, follow-up

## Abstract

**Objectives:** This study aimed to examine the rate of remission in individuals experiencing early-onset poststroke depression (PSD) in China and to identify predictors of remission during a 3-month follow-up. This study also explored the interaction between cognitive impairment and depression.

**Methods:** A total of 820 patients with PSD from a massive multicenter prospective cohort project in China (PRIOD) were included in the present study. Depressive symptoms were measured with the Hamilton Depression Rating Scale (17 Items, HDRS-17) at 2 weeks and the endpoint of the 3-month follow-up. The cut-off score of HDRS-17 (< 8) was used to define remission of depression at the endpoint. The Mini-Mental State Exam (MMSE) was used to evaluate the cognitive impairment of the patients (at the 2-week follow-up and 3-month endpoint). The National Institutes of Health Stroke Scale (NIHSS) was used to measure the severity of stroke.

**Results:** (1) Six hundred and forty-two patients completed the 3-month follow-up, and 332 (51.7%) patients remitted by the end of the study. Univariate analyses indicated that there was a higher proportion of patients who had hypertension, frontal lobe lesion, basal ganglia lesion, poor outcome at 2 weeks, high scores on the NIHSS at 2 weeks, major life events within 3 months, and major medical diseases within 3 months in the nonremission group. In stepwise multiple logistic regression analyses, remission was significantly predicted by lower NIHSS scores at 2 weeks (*p* = 0.001, *OR* = 1.086, 95% CI 1.035–1.139), fewer major life events (*p* = 0.036, *OR* = 5.195, 95% CI 1.111–27.283), fewer major medical comorbidities (*p* = 0.015, *OR* = 2.434, 95% CI 1.190–4.979), and fewer frontal lobe lesions (*p* = 0.042, *OR* = 1.717, 95% CI 1.019–2.891). (2) After controlling for confounding variables, repeated measures analysis of variance revealed a significant interaction between time (2 weeks vs. 3 months) and group (remitters vs. nonremitters) on MMSE scores [*F*_(1, 532)_ = 20.2, *p* < 0.001].

**Conclusions:** Early-onset PSD patients with milder neurological impairment, fewer major life events, fewer major medical comorbidities and no frontal lobe lesion at baseline were more likely to achieve remission 3 months after stroke. Only remitters of PSD improved significantly in cognitive impairment after stroke.

The PRIOD trial is registered at http://www.isrctn.com/, number ISRCTN62169508.

## Introduction

Stroke is still the third leading cause of death, and the incidence rate of stroke has been increasing by 8.7% every year in China (1). However, a progressive decrease in stroke mortality and the subsequent increase of survivors with residual impairments and disabilities have been observed in recent years (2). Stroke can cause physical disability as well as essential emotional and cognitive complications, which can be seriously debilitating. Poststroke depression (PSD) is the most common psychiatric implication of stroke. The prevalence rate is estimated to be 41.8% in the first year following stroke (3), although the rate varies across studies due to methodological discrepancies. It is important to note that depression can result from physical disability (4), and vice versa. Emotional distress can also have a negative influence on the mortality, recovery, physical and cognitive functioning, and quality of life of stroke survivors (5–7).

The mechanisms of PSD are complex and likely to involve multifactorial interactions (8). Previous studies have identified robust predictors of PSD, such as level of functional impairment (4) and stroke severity (9). Sociodemographic factors, such as young/old age, female sex, low education, living alone, a neurotic personality, and unemployment, were often found to be associated with PSD (10–12). Some studies have revealed that patients were more likely to develop PSD if their lesions were on the left side, on the frontal lobe or in the basal ganglia (13–15), but inconsistent findings were also reported (16–18).

To better understand PSD, a few studies have focused on the natural progression and explored trajectories of depressive symptoms following stroke, but the results were far from conclusive (19). PSD cases present in the initial poststroke period may differ from those who develop PSD later, in terms of mechanism and symptomatology. It has been reported that neuroanatomical factors, such as left hemisphere lesions involving the basal ganglia, are responsible for the initial poststroke period depression (20), whereas psychological factors could contribute to both the initial period and later PSD (21). The term “early-onset PSD” is often used to describe the depressive symptoms that appear in the acute phase (within 1–2 weeks after the stroke attack) of stroke (22). Limited attention has been paid to the predictive factors of the remission of early-onset depressive symptoms over time. Therefore, knowledge about the predictors of remission of early-onset PSD is warranted to facilitate clinicians to make an optimal treatment plan, given that administration of antidepressants and nonpharmacological interventions remains controversial (23, 24).

Cognitive impairment after stroke has often been reported with various prevalence rates, possibly due to methodological differences such as the tools and timing of the cognitive evaluation (25, 26). Notably, cognitive impairment after stroke has been associated with reduced functional recovery, increased risk of mortality, and the possibility of evolving to degenerative diseases (27–30). There seems to be a complicated interaction between depressive symptoms and cognitive functioning in poststroke patients over time (31). Cognitive impairments partly overlap with depressive symptoms, and the two syndromes may coexist in patients suffering from stroke (32, 33). In some cases, cognitive impairment caused by stroke may increase the risk of PSD (34, 35); whereas in other cases, cognitive impairment may also result from the depressive symptoms (36). The relationship between PSD and cognitive impairment after stroke has not been sufficiently elucidated in previous studies.

To date, there have been some studies investigating the natural course of PSD, but very few of them specifically focused on early-onset PSD and its interaction with cognitive function over time. This is an important topic that is essential for improving both the long-term physical and psychological outcomes after stroke. The primary aim of this study was to describe the trajectory and outcome of early-onset PSD over a 3-month follow-up, with a focus on remission of depressive symptoms as well as its interaction with cognition. The hypothesis was that patients who remained depressed at the 3-month endpoint would be more likely to have had more severe functional impairments and more risk factors for developing PSD than patients who remitted from their depressive symptoms. The second hypothesis was that the remitters would have more significant cognitive improvements than nonremitters.

## Methods

### Study Participants

The present study was part of a larger project: Incidence and Outcome of patients with poststroke Depression in China (PRIOD) (Project No. ISRCTN62169508, April 2008 to April 2010). PRIOD is a multicenter prospective cohort study with the participation of 56 hospitals (3). The project aimed to investigate the prevalence of PSD in China during a one-year follow-up period after first stroke onset and related risk factors for PSD.

The inclusion criteria of PRIOD were described in detail in a previous publication (3). In brief, patients who fulfilled the following criteria were enrolled in PRIOD: (1) a diagnosis of stroke according to the WHO diagnostic criteria, which was confirmed with CT or MR imaging; (2) onset of stroke within 14 days prior to recruitment; and (3) aged over 18 years old. The exclusion criteria of PRIOD were (1) patients with dementia or other neurological diseases that could affect cognitive functions; (2) patients with a history of or current major psychiatric disorders or alcohol or drug abuse; and (3) patients who did not appropriately communicate.

### Data Collection and Scale Assessment

Eligible patients were consecutively enrolled in the present study. Patients' demographic information, medical history, personal history, family history, diagnostic information, and intervening measures were collected with a case report form at baseline. Major medical comorbidities were defined as cancer, severe cardiovascular disease (acute myocardial infarction, congestive heart failure, angina pectoris), severe kidney disease, and stroke relapse. Major life events were assessed using a self-designed form, and most of the items were adapted from “the list of threatening experiences,” including the death of parents, spouses, or children, as well as serious family conflicts, family members suffering from serious illness, and divorce (37). All these events were commonly reported to cause moderate or marked long-term threat (37).

The stroke patients were screened for depression at 2 weeks after stroke, as was usually done in other studies in early-onset PSD (22, 38). The follow-up assessments were scheduled at 3 months after stroke, because this time point seemed to be a watershed for patients with PSD. Previous studies demonstrated that some biological features of early-onset PSD disappeared at or beyond 3 months after stroke (17, 39).

Experienced neurologists who implemented the scale assessment were blinded to the patients' clinical information. They all received standardized training for the assessments, and interrater reliability reached an acceptable level. Major or minor depression was determined by the Diagnostic and Statistical Manual of Mental Disorders, fourth edition (DSM-IV). DSM-IV suggests that a person should be considered to have mild depressive symptoms if he/she experienced at least 2, but less than 5, of the depressive symptoms listed as the diagnostic criteria for at least 2 weeks, and at least one of the symptoms must be either depressed mood or loss of pleasure/interest (40). The Hamilton Rating Scale for Depression-17 (41) was applied to monitor the degree of depression at the 2-week and 3-month follow-up points. The validity and reliability of the Chinese version HRSD-17 had been proven in previous studies (42). In the present study, early-onset depression was defined as the presence of a depressive episode at 2 weeks after stroke. Remission of depression was defined by the cut-off score on the HRSD-17 (<8) at the endpoint.

The National Institutes of Health Stroke Scale (NIHSS) was used to measure the severity of Stroke (43). The modified RANKIN scale (mRS) was used to assess the neural functional recovery after stroke at the baseline and at the 3-month follow-up point. In the present study, mRS < 2 represented a favorable prognosis (benign outcome), while mRS ≥ 2 indicated an unfavorable prognosis (poor outcome), as in previous studies (44, 45). Cognitive impairment was evaluated with the Mini-Mental State Examination (MMSE) (46). The score for the MMSE scale ranged from 1-30 points. The higher the score, the better the cognitive function.

In the present study, hemorrhagic stroke and ischemic stroke were determined by MRI or CT scan results. The images of MRI (T1 and T2 weighted, fluid-attenuated inversion-recovery sequence, diffusion weighted imaging) or CT scans were retrieved from clinical routine exams. The lesions responsible for the stroke event were identified and reported by radiologists from each site who were blinded to the patients' psychiatric diagnoses. All the research radiologists among various sites have received standard training regarding image interpretation. When the stroke lesions were located in more than one brain region, every affected region was identified, recorded and used in the statistical analyses.

Telephone or face-to-face interviews were conducted at the follow-up point of 2 weeks and 3 months after the stroke attack. Information about the death, stroke relapse, medication regimen, life events, mRS scores, MMSE, and HRSD-17 scores was collected. Antidepressants were prescribed by the treating physicians according to the patients' clinical needs and clinical practice guidelines for depression. Participants were also allowed to take psychotherapy of various types, durations and number of sessions. The use of antidepressants was recorded at 3 months after stroke, which was discussed in detail in our previous article (47).

### Statistical Analysis

Data were analyzed with SPSS 23.0 (SPSS, Inc., IBM Company, USA). Comparisons between remitters and nonremitters with regard to sociodemographic characteristics, scores on the NIHSS and MMSE, functional outcomes as measured by the mRS, lesion locations, medical and personal history with respect to smoking, drinking, taking antidepressants, and receiving psychotherapy were performed using independent sample *t-*tests, Mann–Whitney *U* tests, Fisher's exact test, and chi-square tests, where appropriate. Multivariate logistic regression analyses with the backward Wald method were used to identify predictors of remission at the 3-month follow-up point. In the regression analyses, remission was entered as the dependent variable, and all variables that showed significant differences between the two groups in the aforementioned univariate analyses were entered as independent variables. Receiver operating characteristic (ROC) curves with the area under the curve values were calculated for remission, showing the predicted probabilities from the final model of logistic regression analysis.

Repeated measures analysis of variance (ANOVA) was performed for the Mini-Mental State Examination (MMSE) scores with group (remitters vs. nonremitters) as the between-group factor, time (2 weeks vs. 3 months) as the within-group factor, and variables that showed significant differences between the two groups in the aforementioned univariate analyses as covariates. The effects of time, group, and the interaction between time and group were examined. A two-tailed probability value of *p* < 0.05 was considered to indicate statistical significance.

## Results

**Figure 1:**
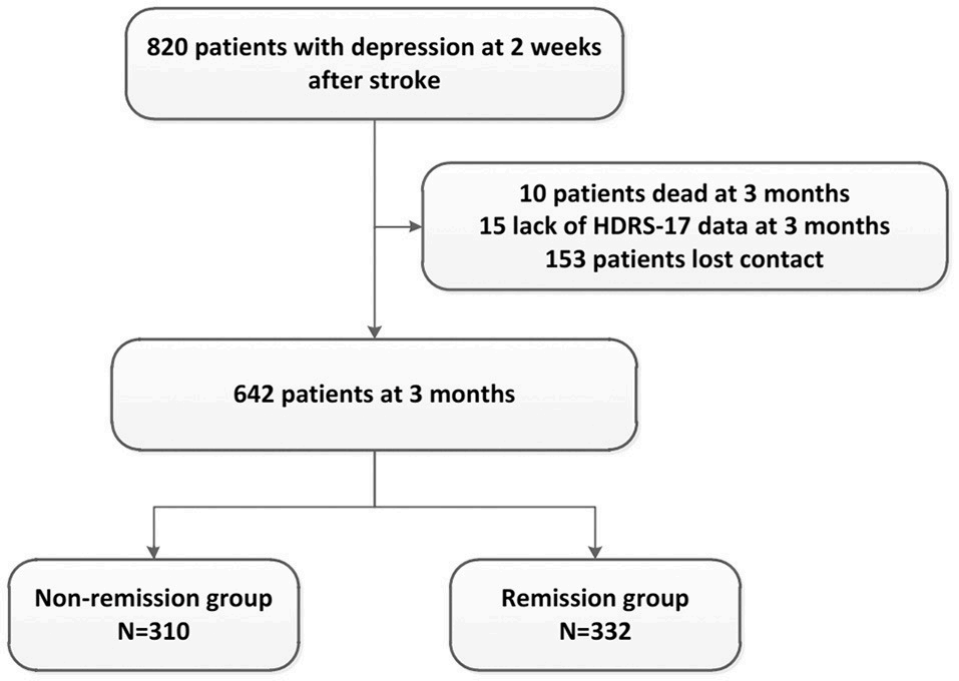
Flow of patients' inclusion and follow-up. HDRS-17, Hamilton Depression Rating Scale (17 items).

Among 2828 patients who participated in PRIOD, 1992 patients were exclude because their HDRS scores were missing or they did not have depression (HDRS-17 total score = < 7) 2 weeks after stroke. Among the remaining 836 patients with depression, 16 patients had a past history of mental disorders. Eight hundred and twenty PRIOD participants met the PSD criteria within 2 weeks after stroke and entered the present study for assessment. Therefore, the prevalence rate of PSD was 29.37% (820/2828). During the follow-up period from the beginning of the 2nd week to the end of the 3rd month, 10 patients died, 153 patients lost contact, and 15 patients lacked HRSD-17 scores. In the end, a total of 642 patients were included in the final analysis. They were divided into the nonremission group (HRSD-17 score ≥ 8, *n* = 310) and the remission group (HRSD-17 score < 8, *n* = 332) according to the HRSD-17 score at the 3-month follow-up (Figure 1).

### Comparison Between Patients Included and Those Excluded From the Analyses

Among 820 patients who entered the study, 178 patients were excluded from the analyses. The excluded patients (*n* = 178) did not differ significantly from the included patients (*n* = 642) with regard to gender (male: 64.04% vs. 59.66%, *p* = 0.289), marital status (married: 89.89% vs. 93.45%, *p* = 0.107), education status (≥12 years of education: 42.24% vs. 35.78%, *p* = 0.183), stroke type (ischemic stroke: 77.97 vs. 77.88%, *p* = 0.795), first episode stroke (68.98 vs. 75.04%, *p* = 0.059), diabetes (28.32 vs. 22.84%, *p* = 0.135), hypertension (68.39 vs. 71.18%, *p* = 0.475), hyperlipidemia (23.57 vs. 19.44% *p* = 0.255), smoking (29.57 vs. 19.44%, *p* = 0.255), drinking (11.24 vs. 12.62%, *p* = 0.620), positive family history of stroke (24.56 vs. 18.50%, *p* = 0.078), stroke relapse (8.33 vs. 2.50%, *p* = 0.134), major life events (0.00 vs. 2.18%, *p* = 1.000), and basal ganglia lesion (47.75 vs. 51.25%, *p* = 0.409). However, there were significant differences in age (64.51 ± 11.67 vs. 61.80 ± 11.54, *p* = 0.006), NIHSS score at 2 weeks (5.25 ± 3.95 vs. 4.08 ± 3.46, *p* < 0.001), MMSE at 2 weeks (23.06 ± 6.36 vs. 24.66 ± 5.26, *p* = 0.006), HDRS-17 at 2 weeks (13.37 ± 4.60 vs. 12.24 ± 3.90, *p* = 0.003), major medical comorbidities (20.83 vs. 6.54%, *p* = 0.022), poor outcome at 2 weeks (67.80 vs. 56.23%, *p* = 0.006), taking antidepressants (62.22 vs. 18.07%, *p* < 0.001), receiving psychotherapy (32.26 vs. 9.03%, *p* < 0.001), and frontal lobe lesion (20.22% vs. 11.06%, *p* < 0.001) compared to the patients included in the analysis. The two groups of patients were compared in order to determine whether the patients included in the following analyses were representative of the whole sample, and whether the conclusion could be generalized to other populations.

### Comparison of Demographic and Clinical Characteristics Between Remitters and Non-remitters at 3 Months After Stroke

At the end of the 3-month follow-up, there were 332 (51.7%) remitters and 310 (48.3%) nonremitters determined by the cut-off point of the HDRS-17 total score. The average total score on the HDRS-17 was 13.33 ± 4.63 in nonremitters and 3.89 ± 2.14 in the remitters. The baseline demographic and clinical characteristics of remitters and nonremitters at the 3-month follow-up are presented in Table 1. Univariate analyses indicated that there was a higher proportion of patients with hypertension (74.92 vs. 67.69%, *p* = 0.046), frontal lobe lesion (13.87 vs. 8.44%, *p* = 0.028), basal ganglia lesion (55.48 vs. 47.29%, *p* = 0.038), poor outcome at 2 weeks (61.29 vs. 51.51%, *p* = 0.013), high scores on NIHSS at 2 weeks (4.61 ± 3.67 vs. 3.59 ± 3.18, *p* < 0.001), major life events within 3 months (3.87 vs. 0.60%, *p* = 0.005), and major medical comorbidities at 3 months (9.68 vs. 3.61%, *p* = 0.002) in the nonremission group. On the other hand, remitters had a low HDRS-17 total score (11.42 ± 3.32 vs. 13.11 ± 4.27, *p* < 0.001) at 2 weeks.

**Table 1:** Comparison of demographic and clinical characteristics between remitters and nonremitters at 3 months.

**Variables**	**Remitters (*n* = 332)**	**Nonremitters (*n* = 310)**	***X*^**2**^**	***p*-value**
**DEMOGRAPHIC CHARACTERISTICS**				
Age (mean ± SD)	61.67 ± 11.64	61.93 ± 11.45	–	0.734
Male (%)	61.45	57.74	0.914	0.339
Married (%)	93.05	93.87	0.176	0.675
Education ≥ 12 years (%)	36.56	34.95	0.179	0.672
**VASCULAR RISK FACTORS**				
Family history of stroke (%)	19.69	17.26	0.610	0.435
Smoking (%)	32.65	30.49	0.335	0.563
Drinking (%)	13.86	11.29	0.957	0.328
Diabetes (%)	20.68	25.17	1.785	0.182
Hypertension (%)	67.69	74.92	3.990	0.046^*^
Hyperlipidemia (%)	20.47	18.32	0.422	0.516
**NEUROIMAGING CHARACTERISTICS**				
Frontal lobe (%)	8.44	13.87	4.818	0.028^*^
Temporal lobe (%)	12.05	9.35	1.212	0.271
Parietal-occipital lobe (%)	15.96	14.52	0.260	0.610
Basal ganglia (%)	47.29	55.48	4.309	0.038^*^
Infratentorial region (%)	24.40	19.35	2.379	0.123
Left side lesion	34.91	36.21	0.115	0.734
**CLINICAL VARIABLES**				
Ischemic stroke (%)	79.82	75.80	1.499	0.221
First episode of stroke (%)	77.34	72.58	1.937	0.164
Poor outcome at 2 weeks (%)	51.51	61.29	6.236	0.013^*^
Taking antidepressants (%)	16.27	20.00	1.511	0.219
Receiving psychotherapy (%)	7.23	10.97	2.727	0.099^*^
Major life events (%)	0.60	3.87	8.029	0.005^*^
Major medical diseases (%)	3.61	9.68	9.638	0.002^*^
Stroke relapse (%)	2.11	2.90	0.409	0.523
NIHSS at 2 weeks (mean ± SD)	3.59 ± 3.18	4.61 ± 3.67	–	<0.001^*^
MMSE at 2 weeks (mean ± SD)	24.95 ± 4.89	24.31 ± 5.67	–	0.334
HDRS at 2 weeks (mean ± SD)	11.42 ± 3.32	13.11 ± 4.27	–	<0.001^*^
MMSE at 3 months (mean ± SD)	26.63 ± 3.65	24.72 ± 5.31	–	<0.001^*^
HDRS at 3 months (mean ± SD)	3.89 ± 2.14	13.33 ± 4.63	–	–

*SD, Standard Deviations; NIHSS, National Institutes of Health Stroke Scale; MMSE, Mini-Mental Exam; HRSD-17, Hamilton Depression Rating Scale (17 items)*;

* *P <0.05*.

### Exploring the Independent Predictors of Remission at 3 Months After Stroke

**Figure 2:**
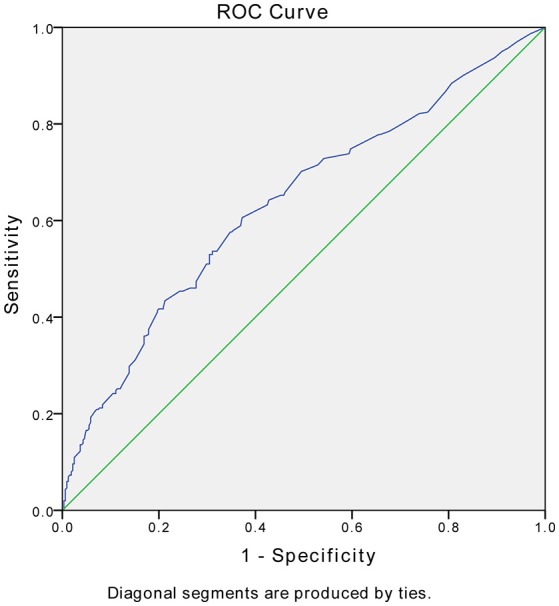
Receiver operating characteristic (ROC) curves for the model of remission vs. nonremission. The area under the ROC curve was estimated to be 0.637 (*p* < 0.001, 95% CI 0.593–0.680).

Stepwise multivariate logistic regression analyses were used to identify predictors of remission of PSD at 3 months. In the regression analyses, remission was entered as the dependent variable, and all variables that showed significant differences between two groups in the aforementioned univariate analyses were entered as independent variables. The results are shown in Table 2. Remission was significantly predicted by lower NIHSS scores at 2 weeks (*p* = 0.001, *OR* = 1.086, 95% CI 1.035–1.139), fewer major life events (*p* = 0.036, *OR* = 5.195, 95% CI 1.111–27.283), fewer major medical comorbidities (*p* = 0.015, *OR* = 2.434, 95% CI 1.190–4.979), and not having frontal lobe lesions (*p* = 0.042, *OR* = 1.717, 95% CI 1.019–2.891). Figure 2 presents the ROC curve for the predicted probabilities from the final model of the multiple logistic regression analysis. The area under the ROC curve was estimated to be 0.637 (*p* < 0.001, 95% CI 0.593–0.680), indicating that the overall accuracy of the final model to predict patients' remission (with a predicted probability of 0.5 or greater) was acceptable.

**Table 2:** Predictors of nonremission at 3 months (multivariate stepwise logistic regression model) (*n* = 642).

	**B**	**S.E**.	**Wald**	**Sig**.	**OR**	**95% C.I**.	
						**Lower**	**Upper**
NIHSS at 2 weeks	0.082	0.025	14.298	0.001	1.086	1.035	1.139
Major life events	1.648	0.787	4.368	0.036	5.195	1.111	24.283
Major medical comorbidities	0.890	0.365	5.938	0.015	2.434	1.190	4.979
Frontal lobe lesion	0.540	0.266	4.128	0.042	1.717	1.019	2.891
Basal ganglia lesion	0.283	0.168	2.829	0.093	1.328	0.954	1.847
Hypertension	0.313	0.184	2.902	0.088	1.367	0.954	1.960

### Comparison Between Remitters and Nonremitters Regarding Longitudinal Changes in MMSE

**Figure 3:**
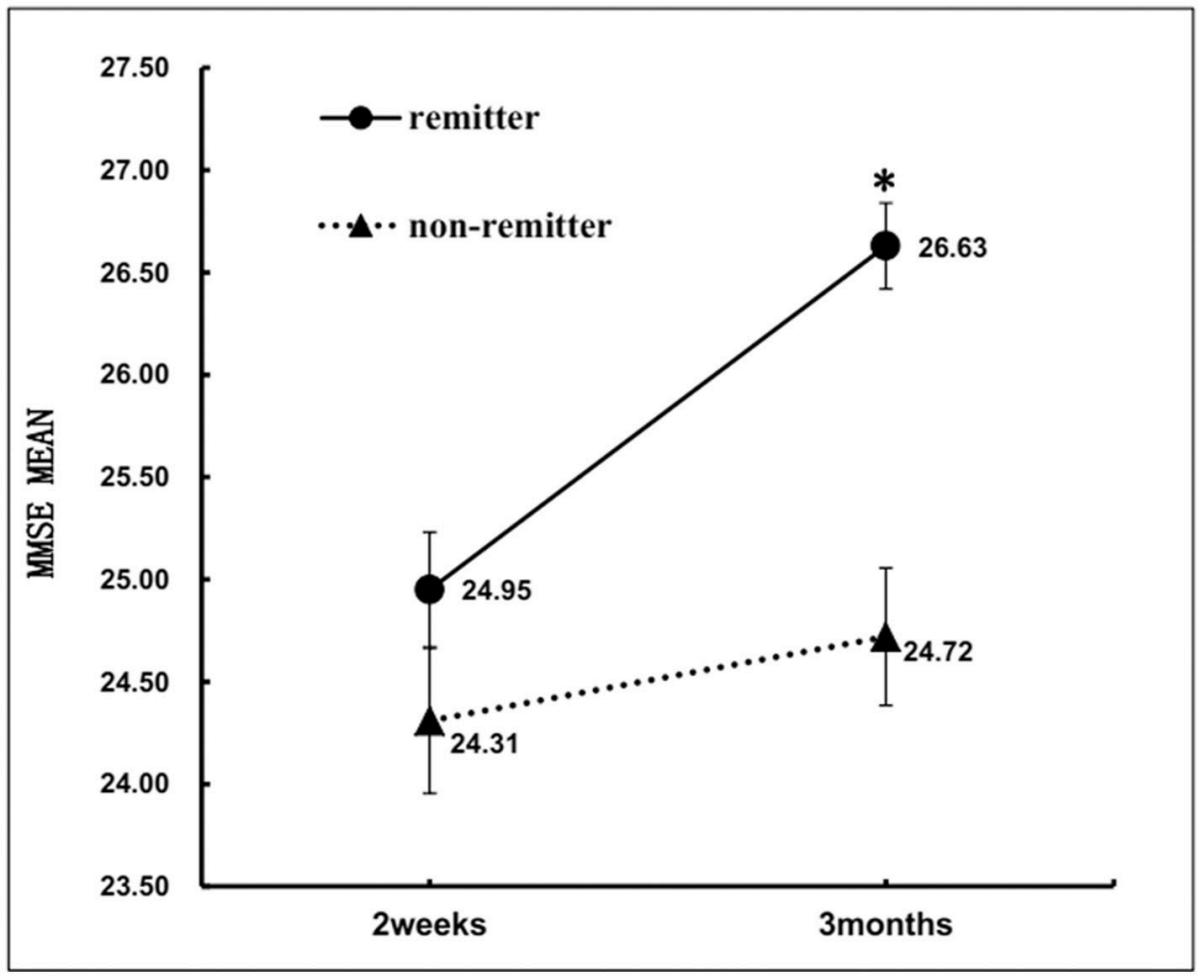
Changes in MMSE scores over follow-up time in remitters and nonremitters. *p < 0.001.

At 2 weeks after stroke, there was no significant difference in MMSE between the two groups. However, nonremitters performed significantly poorer on MMSE than remitters at 3 months after stroke (Table 1). After controlling for NIHSS, hypertension, major life events, major medical comorbidities, frontal lobe lesion and basal ganglia lesion, the results of a repeated measures ANOVA revealed significant time (2 weeks vs. 3 months) ^*^group (remitters vs. nonremitters) interaction on MMSE [*F*_(1, 532)_ = 20.2, *p* < 0.001]. In the remitter group, MMSE scores changed toward better performance from 2 weeks (24.95 ± 4.89) to 3 months (26.63 ± 3.65). In the non-remitter group, MMSE scores did not change from 2 weeks (24.31 ± 5.67) to 3 months (24.72 ± 5.31) (Figure 3).

## Discussion

This study systematically examined predictors of clinical remission of early-onset PSD and explored the potential interaction between depressive symptoms and cognitive impairment after stroke. The two hypotheses were both confirmed by the results. Milder neurological impairment as indicated by NIHSS at 2 weeks, fewer major life events, fewer medical comorbidities and no frontal lobe lesion were significant predictors of the remission of PSD 3 months after stroke. These factors have been reported in previous studies as risk factors for the development of PSD (15, 48). For early-onset PSD patients, only remitters gained significant improvement in cognition over 3 months of follow-up.

Among the patients from PRIOD, 29.37% had depressive symptoms. This prevalence rate of PSD is close to most of the rates reported in previous studies, in which PSD was generally observed in approximately one-third of stroke cases, despite a large variety of criteria used to diagnose PSD (49–52). Among depressed patients who completed the 3-month follow-up, the remission rate was 51.7%. Some authors believe that the symptoms of PSD are self-limiting, and a longitudinal study reported that most patients diagnosed with acute PSD recovered (53). Previous studies demonstrated that approximately 50% of PSD patients would have symptom remission after 6 months, and the rate would increase to 89% at 12 months after stroke (54). Remission of PSD over the first a few months after stroke is vital, and symptom remission has been associated with higher recovery in the activity of daily living function for these patients (55).

The logistic regression analysis in the present study clearly confirmed that the course of PSD was a result of multifactorial interactions involving both biological and psychosocial determinants. Stroke severity and lesion locations served as the main biological factors in this study. Stroke severity, as indicated by NIHSS scores, has been one of the most consistent risk factors for PSD. A growing body of evidence has shown that the more severe the stroke is, the more likely a patient would develop PSD (26, 56). Higher NIHSS scores were associated with more severe depression after stroke. The results from Ilut et al.'s study suggested that the NIHSS score can predict the long-term prognosis of stroke, and a score over 11 could even bring a 9.4-fold higher probability of experiencing severe depression (15). This association may represent the biological mechanism of the pathophysiology of PSD. Severe stroke could lead to a series of biological changes in the brain and body, as well as psychological and functional alterations, all of which could contribute to the development of depression. Serious brain lesions may damage the function of some brain regions that are responsible for mood (57, 58). Much attention has been paid to the relationship between the onset of PSD and lesion locations, although this issue remains inconclusive (59, 60). Frontal lobe lesions were identified as a predictor of nonremission, which is in line with previous clinical and laboratory studies. Researchers have been trying to establish a connection between neuroimaging markers and the occurrence and development of depressive symptoms after stroke. Some neural circuits have been implicated in the development of both major depressive disorder and PSD (61–65), often involving frontal areas (51, 66, 67). On the one hand, the frontal lobe plays a critical role in regulating emotion and cognitive functions (68). On the other hand, metabolic changes were discovered in the frontal lobe in PSD patients through MRI spectroscopy (69). Experimental rats with middle cerebral artery occlusion (MCAO) were found to be 14 times more likely to exhibit depressive-like behaviors than sham-operated control rats, and BDNF levels were downregulated in certain brain regions in the frontal and other cortical regions (70). The prefrontal cortex has also been implicated in the bilateral internal carotid artery occlusion (BICAO) model as one of the several vulnerable brain areas associated with depressive-like behaviors after ischemia (71). PSD patients with frontal lobe lesions exhibited more persistent or recurrent symptoms than those without frontal lobe lesions in the first year after stroke onset (72).

Moreover, the stroke attack may also result in a decreased socioeconomic status, quality of life and general self-efficacy (GSE) (73), which contributed to a vicious circle between functional deficits and onset of PSD (74, 75). Other psychosocial factors include suffering from major medical comorbidity and exposure to major life events, which were also independent predictors of nonremission. Patients who underwent severe major medical diseases (including stroke relapse) after stroke attack may have an increased risk of depression onset or deterioration. Comorbid medical conditions may result in a patient's increased psychological burden, including a decline in the quality of life and rehabilitation faith, which may further cause depressed mood (76). Then, the depressed mood may in turn negatively affect the existing medical conditions, thus creating a vicious circle (77). Even in depressive patients who had no previous stroke, somatic symptoms have been reported to potentially worsen the outcome of depressive disorder. A study in patients with major depressive disorder indicated that remission rates in patients with more severe somatic symptoms were significantly lower than those in patients without somatic symptoms (78). Another 2-year follow-up study showed that “somatic symptoms” was an independent predictor of a worse prognosis of MDD (79). Therefore, major medical comorbidities and their companying somatic symptoms may have a tremendous negative impact on the probability of remission of depression. In the general population, exposure to major life events is a risk factor for the subsequent development of the depressive disorder (80). Likewise, studies demonstrated that patients with PSD had more major life events than nondepressed stroke patients before and after 6 months of stroke onset (48, 81, 82). The present study found that major life events not only served as a risk factor for developing depression but also prevented PSD patients from achieving remission. Therefore, more medical attention should be paid to PSD patients experiencing major life events.

Hypertension and poor outcomes at week 2 showed statistical significance in the univariate analysis, but they were eventually removed in the multivariate logistic regression. Concerning hypertension, the present result suggested that it did not contribute to the symptom resolution of depression, which is consistent with previous literature (83). The chi-square test indicated that mRS scores were significantly lower in the remission group than in the nonremission group. Many studies have shown that functional impairments might play a key role in the pathogenesis and development of PSD (16, 54). In a review in 2014, the researchers revealed that depression was negatively associated with functional outcome in stroke survivors and that the severity of stroke was the most significant contributor to PSD (84). However, a poor functional outcome may also be due to stroke severity and major medical comorbidities, and these are more robust independent predictors of remission in the present study.

The beneficial effects of antidepressant treatment in patients with PSD have been proved in a number of previous studies. These studies suggested noradrenaline reuptake inhibitors (NRIs), selective serotonin reuptake inhibitors (SSRIs), and tricyclic antidepressants (TCAs) all brought a considerable higher HAMD score reduction than the control treatments (85). There has also been a body of evidence indicating positive effects of cognitive behavioral psychotherapy in patients with PSD (86). However, neither antidepressants nor psychotherapy was associated with higher remission rate in the present study. PRIOD is a non-interventional study. Antidepressants and psychotherapies were used according patient's clinical needs with various doses, regimens, durations and number of sessions. These confounding factors could partly explain the inconsistent results with previous studies.

The present study also found a significant group (remitters vs. nonremitters) by time (from 2 weeks to 3 months) interaction with respect to MMSE scores, and only remitters had a significant improvement on the MMSE. Cognitive impairment and its relationship with depression in stroke survivors have long been of interest to the research community. Ischemic brain injury can cause both dementia (87) and depression (56). However, the causal relationship between cognitive impairment and depression after stroke remains debatable. In some studies, significant improvement had been achieved with regard to poststroke depressive symptoms during treatment with antidepressants, while cognitive function remained impaired (88–90). Therefore, some authors claimed that depressive symptoms might be secondary to cognitive impairment, which was caused by stroke and would follow its own course of recovery (88). However, cognitive functioning did not improve significantly in the aforementioned studies may be attributed partly to the inclusion of mixed cohorts of patients with various severities of depression. Patients with mild depressive disorder would not be expected to show cognitive improvement (32, 91). In Murata et al.'s study, only patients with major depressive disorder were enrolled, and patients with a significant reduction in depression severity also showed significant cognitive improvement over time (32). Although the present research also included both major and mild depression, as described in the method section, the outcome measure is symptom remission rather than reduction of depression severity. In addition, the sample size of the present study is much larger than that of previous studies. Therefore, the discrepancies with some previous studies could possibly be due to these methodological differences. The interaction between cognitive function and depressive symptoms in patients with PSD warrants further exploration.

The strengths of the present study include a large sample size, a wide range of sociodemographic and clinical variables, and the exclusion of patients with a history of mental disorders. However, the results should be explained with caution due to the following methodological limitations. First, 21.7% patients were excluded from the analyses because of incomplete follow-up information, and they were significantly different from the included patients in some factors. For this reason, the conclusion could not be simply applied to all studied populations. Second, patients with dementia and severe aphasia were excluded from the present study, and the subjects enrolled had relatively moderate deficits in neurological function and lower mortality. The exclusion of patients with severe cognitive and neurological deficits may potentially prevent generalization of findings to all stroke patients. Third, the MMSE has been found to overestimate impairments in persons over age 60 and in persons with less than 9 years of education (92); thus, it may not be sensitive to the cognitive changes in the present sample. A more sensitive and detailed neuropsychological battery is needed to monitor the longitudinal changes in cognitive function in patients with early-onset PSD. Fourth, psychological determinants, such as personality and coping styles, also play an important role in the course of PSD, but they were not collected in the present study.

## Conclusion

In conclusion, this study showed that approximately half of early-onset PSD patients remitted 3 months after stroke. Patients with less severe stroke, fewer major life events, fewer major medical comorbidities and frontal lobe lesions were more likely to have a favorable outcome regarding depression 3 months after stroke. Moreover, only remitters of PSD improved significantly in cognitive impairment after stroke. These results highlight the importance of early identification and intervention for patients with potentially persistent depression after stroke.

## Data Availability Statement

The datasets analyzed for this study can be found in the website: http://www.tt.zhinanmed.com/.

## Ethics Statement

The PRIOD protocol was approved by the Medical Ethics Committee of Beijing TianTan Hospital, Capital Medical University. The project was carried out in accordance with the Declaration of Helsinki Guidelines, and all participants offered written consent form for the study.

## Author Contributions

JH and F-CZ wrote the draft of the manuscript. JH, F-CZ, BG, PY, and LZ organized the database. AW and F-CZ performed the statistical analysis. C-YW and CW contributed the revision of the final version. CW contributed conception and design of the study. All authors contributed to manuscript revision, read and approved the submitted version.

### Conflict of Interest Statement

The authors declare that the research was conducted in the absence of any commercial or financial relationships that could be construed as a potential conflict of interest.
